# A Comparison Study of Person Identification Using IR Array Sensors and LiDAR

**DOI:** 10.3390/s25010271

**Published:** 2025-01-06

**Authors:** Kai Liu, Mondher Bouazizi, Zelin Xing, Tomoaki Ohtsuki

**Affiliations:** 1 Graduate School of Science and Technology, Keio University, Yokohama 223-8522, Japan; k.liu@ohtsuki.ics.keio.ac.jp (K.L.); xingzelin@ohtsuki.ics.keio.ac.jp (Z.X.); 2 Faculty of Science and Technology, Keio University, Yokohama 223-8522, Japan; bouazizi@ohtsuki.ics.keio.ac.jp

**Keywords:** person identification, deep learning, IR array sensor, LiDAR

## Abstract

Person identification is a critical task in applications such as security and surveillance, requiring reliable systems that perform robustly under diverse conditions. This study evaluates the Vision Transformer (ViT) and ResNet34 models across three modalities—RGB, thermal, and depth—using datasets collected with infrared array sensors and LiDAR sensors in controlled scenarios and varying resolutions (16 × 12 to 640 × 480) to explore their effectiveness in person identification. Preprocessing techniques, including YOLO-based cropping, were employed to improve subject isolation. Results show a similar identification performance between the three modalities, in particular in high resolution (i.e., 640 × 480), with RGB image classification reaching 100.0%, depth images reaching 99.54% and thermal images reaching 97.93%. However, upon deeper investigation, thermal images show more robustness and generalizability by maintaining focus on subject-specific features even at low resolutions. In contrast, RGB data performs well at high resolutions but exhibits reliance on background features as resolution decreases. Depth data shows significant degradation at lower resolutions, suffering from scattered attention and artifacts. These findings highlight the importance of modality selection, with thermal imaging emerging as the most reliable. Future work will explore multi-modal integration, advanced preprocessing, and hybrid architectures to enhance model adaptability and address current limitations. This study highlights the potential of thermal imaging and the need for modality-specific strategies in designing robust person identification systems.

## 1. Introduction

Person identification is a fundamental task in biometrics and computer vision, with applications covering security, surveillance, and access control. Unlike person re-identification [1], which focuses on matching individuals across different scenes or camera views, person identification [2] determines the identity of an individual from a database using input data. This task often requires robust feature extraction methods to handle challenges such as lighting variations, occlusions, and complex environments. Various types of data have been utilized for person identification, including RGB images, thermal images, depth data, and physiological signals such as heartbeat [3] and respiration [4], each accommodating specific application scenarios and environmental conditions. In this study, we explore the effectiveness of three data modalities—RGB images, thermal images, and depth images—for person identification, analyzing their performance under varying resolutions and conditions.

### 1.1. Background

Person identification has been extensively studied in the fields of biometrics [5] and computer vision [6], driven by its critical role in applications such as security surveillance [7], access control [8], and public safety [9]. The task typically involves distinguishing individuals based on input data, mostly using RGB images. Very few works used depth and thermal images. Each modality presents unique advantages and challenges, making it essential to evaluate and compare their effectiveness in real-world scenarios.

Table 1 shows the advantages and disadvantages of Millimeter Wave (mmWave) radar, RGB, thermal, and depth imaging for person identification. Given their different capabilities, each of these techniques has its merits and is best suited for specific scenarios and environmental conditions that fit with their capabilities.

Millimeter wave radar performs well in scenarios with occlusions or poor visibility, such as fog, smoke, or privacy-sensitive environments. It remains unaffected by lighting conditions and changes in appearance, focusing instead on skeletal movements and body shapes. However, its limited spatial resolution reduces its effectiveness in distinguishing fine details, and it is susceptible to electromagnetic interference.

RGB imaging is highly advantageous in well-lit environments, providing detailed texture and color information for accurate identification. Despite its widespread use, it struggles in low-light conditions or cluttered backgrounds. The models often rely on non-robust features, such as high-contrast clothing elements, making RGB imaging highly sensitive to changes in appearance.

Thermal imaging offers robust performance in low-light and nighttime conditions, relying on heat signatures to identify individuals while preserving privacy. Its focused attention on person-specific features, such as the head and torso, enhances reliability in challenging environments. However, overlapping thermal profiles and background heat artifacts can reduce its accuracy, particularly when multiple heat sources are present.

Depth imaging captures three-dimensional structural features, enabling identification based on body shape. It is relatively resilient to changes in clothing or surface appearance but suffers from noise, artifacts, and incomplete data, particularly at lower resolutions. Its performance further diminishes in dynamic environments or when background clutter interferes with depth perception.

This comparative analysis highlights the complementary strengths of these modalities, highlighting the robustness of millimeter wave radar in occluded environments and the adaptability of thermal imaging in challenging scenarios. Depth and RGB data offer valuable insights in controlled settings but require advanced preprocessing and model refinements to overcome their inherent limitations.

Identifying the person using RGB images is one of the most widely studied modalities [10,11,12,13]. It is effective in well-lit conditions and provides detailed color and texture information. However, this method is highly sensitive to lighting variations, occlusions, and cluttered environments. These challenges can significantly impact the accuracy of person identification systems. The survey [12] highlights the limitations of RGB-based person identification, especially under varying lighting conditions and occlusions. Depth images, obtained through sensors such as LiDAR or structured light systems, provide geometric and structural data, enabling robust performance under certain conditions. Research like Zhu et al. [14] and Shao et al. [15] highlighted the potential of depth-based identification systems in environments where shape information is critical. Thermal imaging captures the heat signatures emitted by individuals, making it robust against lighting variations and effective in low-visibility scenarios. Unlike visible light cameras, thermal cameras detect infrared radiation, allowing them to function effectively in darkness, smoke, and fog. The study [16] highlights how thermal imaging enhances recognition accuracy, especially under challenging lighting conditions. However, the study [17] shows that when the background temperature is similar to that of the human body, distinguishing individuals becomes challenging, which reduces the effectiveness of thermal imaging.

Previous studies have also explored multimodal approaches to use the complementary strengths of these modalities. For example, Hafner et al. [18] proposed combining RGB and depth data for enhanced person re-identification. Shopovska et al. [19] utilized thermal data alongside RGB to improve robustness in low-light environments. Despite these advances, challenges such as overfitting, reliance on background details, and scalability to real-life conditions persist.

Some applications of millimeter wave radar to person identification relies on gait features. In our study, which focuses on RGB, thermal, and depth imaging, we emphasize that our approach fundamentally differs from mmWave sensors as we do not rely on gait features. Instead, we focus on capturing and analyzing actual physical features of the human body, such as shape, texture, heat distribution, and three-dimensional structure.

Specifically, RGB imaging uses high-resolution visual features like skin texture, and facial details which are extracted by deep learning models for classification. Thermal imaging provides heat signature data, isolating key physiological areas such as the head, chest, and limbs, independent of appearance or clothing. Depth imaging contributes by capturing the subject’s three-dimensional structure, such as body shape and posture, which is robust against visual noise and appearance changes. Regarding the sensitivity of the subject’s orientation relative to the sensor, while gait features play a critical role in mmWave radar-based systems, orientation is less critical in our multimodal setup.

For example, RGB data is most effective when the subject faces the sensor, allowing for full exposure of visual features, though it can still operate under slight angular variations. Thermal data is less dependent on orientation, as heat signatures are captured uniformly, provided that the critical regions (head, chest, etc.) are within the field of view. Depth data works best when the subject is within a certain range and orientation, as occlusions or extreme angles can affect depth accuracy.

Our study does not explicitly analyze gait-related features but instead focuses on identifying subjects based on these physical attributes in controlled walking scenarios. This approach provides a more generalized framework for person identification that can be applied in diverse settings, including those where gait features are either unavailable or unreliable. Additionally, while our study confines subjects to predefined paths for consistency, future research could explore more dynamic, unconstrained scenarios to assess the robustness of these modalities in natural settings.

In this work, we extend the current research by comparing the performance of RGB, thermal, and depth modalities for person identification using a dataset collected in diverse, real-life scenarios. Unlike most existing studies that focus on person re-identification, our focus is on identification, emphasizing the generalizability of each modality. Through heatmap analysis and quantitative evaluations, we investigate the strengths and limitations of these modalities, particularly their ability to focus on subject-related features rather than irrelevant background details.

### 1.2. Motivations

The demand for accurate and robust person identification systems has grown rapidly, driven by critical applications in security, forensics, and intelligent monitoring. While advances in computer vision and deep learning have elevated identification performance, real-world deployment still faces persistent challenges. Environmental variability, overfitting, and the reliance on specific data modalities often hinder the reliability and generalizability of existing systems.

Among commonly used modalities, RGB images dominate due to their accessibility and detailed visual information. However, their efficacy is limited in low-light environments and cluttered backgrounds, where irrelevant features may confuse models. Depth images offer complementary 3D structural insights and are resilient to color variations, yet they are prone to the background in lower resolution conditions. Thermal imaging emerges as a powerful alternative, providing lighting-independent heat signatures that remain robust across challenging scenarios. Despite this, thermal-based person identification remains underexplored compared to RGB and depth modalities.

This study is motivated by the need to bridge these gaps through a comprehensive evaluation of RGB, depth, and thermal imaging. By integrating these modalities within a unified framework, we aim to analyze their individual and collective strengths under realistic, diverse environmental conditions. Through heatmap analyses, we further seek to ensure models prioritize meaningful subject features over irrelevant background details. This research aims to advance the design of resilient, multimodal identification systems customized to real-world operational demands.

### 1.3. Contribution

This study presents a comprehensive evaluation of RGB, thermal, and depth modalities for person identification, analyzing their strengths and limitations across diverse resolutions and real-world scenarios. Through performance benchmarking and heatmap visualizations, we demonstrate that thermal imaging outperforms RGB and depth imaging in maintaining focus on subjects rather than irrelevant background details, showcasing its robustness under challenging conditions.

To support this evaluation, we introduce a novel dataset collected in varied and realistic environments, designed to simulate practical conditions. This dataset enables a detailed exploration of modality-specific challenges and highlights the potential of thermal imaging as a reliable alternative for consistent identification in low-visibility or cluttered scenarios.

Our findings provide actionable insights for the design of robust person identification systems, emphasizing the need for feature generalization and the integration of thermal imaging to address challenges posed by environmental variability. By bridging the gap between modality-specific limitations and practical requirements, this study contributes to advancing multimodal identification frameworks suited for diverse operational environments.

## 2. Related Work

Person identification is a fundamental task in biometrics and computer vision, with applications ranging from security and surveillance to forensic investigations.

Over the years, person identification has relied mostly on RGB images due to their being easier to collect, and due to the availability of large-scale datasets created and published. Deep learning models, such as convolutional neural networks (CNNs) and Vision Transformers (ViTs), have been effectively applied to extract identity-specific features from RGB images in well-lit environments [20]. Studies such as Kowalski et al. [21] demonstrated the efficacy of CNN-based models for feature extraction, while Zhao et al. [22] explored attribute-driven feature disentangling for robust classification. However, RGB models often degrade under low-light conditions or in cluttered environments, emphasizing the need for complementary modalities or advanced preprocessing techniques. YOLO [23] is a widely used object detection framework. It has been integrated into person identification pipelines to crop irrelevant areas and improve focus on subjects.

Depth imaging offers the advantage of capturing three-dimensional structural information, making it possible to distinguish individuals based on body shape and spatial features. Patruno et al. [14] highlighted the potential of multimodal approaches combining depth with RGB and skeleton data for people re-identification. However, Chen et al. [24]. revealed that depth imaging remains sensitive to noise and performs poorly in uncontrolled conditions, particularly at lower resolutions. These limitations underscore the importance of preprocessing and denoising techniques to enhance depth data reliability.

Thermal imaging has gained attention for its ability to capture heat signatures that are invariant to lighting conditions, making it highly effective in low-light or nighttime scenarios. Studies like that of Kowalski et al. [21] demonstrated the robustness of thermal imaging for person identification, with models utilizing unique body temperature patterns for classification. Nonetheless, thermal imaging faces limitations, such as reduced effectiveness in environments where the background temperature closely matches human body temperature, leading to decreased thermal contrast and identification accuracy [25].

Recent research has increasingly explored multimodal approaches, combining RGB, thermal, and depth modalities for person identification tasks, as they effectively utilize the complementary strengths of different data types [26,27,28]. By integrating data from RGB, depth, and thermal modalities, these systems enhance robustness and adaptability across varying conditions such as low lighting, occlusions, or environmental complexities. The study by Wu et al. [29] explores the integration of RGB and thermal data. Although primarily focused on re-identification, the insights offered on handling diverse data sources are directly applicable to person identification tasks. The authors introduce techniques for effective fusion and matching of RGB and IR features, which could be extended to identification scenarios where both modalities are available. This highlights the potential of IR imaging to complement RGB data, particularly in nighttime or poorly lit conditions. Similarly, the survey by Uddin et al. [12] provides an extensive overview of how multimodal systems integrate RGB, depth, and IR data. It emphasizes the importance of combining these data types to address limitations inherent to single-modality approaches. The survey outlines key techniques for sensor fusion and feature alignment, making it a valuable resource for researchers designing multimodal identification systems. While both works contribute valuable insights into multimodal data integration, they do not fully address the specific challenges of person identification, such as how to integrate diverse data sources effectively for robust identification in dynamic, real-world environments.

Our work addresses this gap by conducting a detailed comparative analysis of RGB, thermal, and depth modalities specifically for person identification. By using YOLO-based preprocessing and analyzing heatmaps generated through GradCAM [30] (for ResNet34 [31]) and Attention Rollout [32] (for ViT [33]), this study provides a deeper understanding of the strengths and limitations of each modality in practical applications.

## 3. Prelimiaries

The data collection for this study utilized three primary sensor technologies: infrared (IR) array sensors for thermal imaging, LiDAR for depth imaging, and typical commercial cameras for RGB imaging. In our work, we use two devices equipped with these 3 sensing technologies: a thermal device equipped with both an IR array sensor and an RGB camera, and a depth device equipped with both a LiDAR and an RGB camera.

### 3.1. IR Array Sensors

IR array sensors are widely used for thermal imaging due to their ability to capture infrared radiation emitted by objects as heat [34]. These sensors produce thermal images that are independent of lighting conditions, making them ideal for applications in low-light or nighttime scenarios. The thermal images collected in this study provide heat signature data that can highlight unique features of individuals, such as body shape and temperature patterns, which are invariant to changes in illumination. This capability ensures robustness in challenging environments where traditional RGB imaging might fail.

### 3.2. LiDAR

Light Detection and Ranging (LiDAR) [35] technology was employed to capture both RGB and depth data. LiDAR sensors use laser pulses to measure distances, generating highly accurate three-dimensional spatial information. The RGB images collected by the LiDAR system offer detailed visual information, including color and texture, which are essential for identifying individuals under favorable lighting conditions. Additionally, the depth images generated by LiDAR provide geometric and structural data, allowing models to distinguish individuals based on shape and spatial features. These complementary capabilities make LiDAR an excellent tool for multimodal data collection.

### 3.3. Deep Learning for Computer Vision

Deep learning has revolutionized the field of computer vision, enabling state-of-the-art performance in tasks such as image classification [36], object detection [37], and semantic segmentation [38]. Central to these advancements are powerful architectures designed to learn complex representations from data and interpretation methods that provide insights into model decisions. This subsection focuses on the models and interpretation methods used in this study: ViT [33], ResNet34 [31], Attention Rollout [32], and GradCAM (Gradient-weighted Class Activation Mapping) [30].

#### 3.3.1. ViT

Introduced by Dosovitskiy et al., the ViT [33] applies a transformer architecture, originally developed for natural language processing [39], to image classification. ViT divides an image into fixed-size patches, encodes them into embeddings, and processes them using multi-head self-attention mechanisms. This design enables ViT to capture long-range dependencies and achieve competitive performance on large datasets.

#### 3.3.2. ResNet34

ResNet34 [31] is a convolutional neural network (CNN) that introduced the concept of residual connections to mitigate the vanishing gradient problem in deep networks [40]. These residual connections allow the network to learn identity mappings, enabling deeper architectures without performance degradation. ResNet34, with its 34-layer design, is widely used for image classification due to its balance between depth and computational efficiency.

#### 3.3.3. Attention Rollout

Attention Rollout [32] is an interpretation method designed for transformer-based models such as ViT. It aggregates the attention weights from all layers to visualize how information flows through the model, revealing the regions of the input image that contribute most to the classification decision. This method is particularly effective in understanding the spatial relationships learned by ViT.

#### 3.3.4. GradCAM

GradCAM [30] is a popular visualization technique for CNN-based models, such as ResNet34. It highlights the regions of an input image that influence a model’s prediction by using the gradients of the target class with respect to the feature maps in the last convolutional layer. GradCAM provides intuitive heatmaps, making it a widely used tool for understanding CNN-based classification.

## 4. System Description and Setup

### 4.1. System Description

The proposed system aims to provide a secure, contactless, and resource-efficient solution for person identification at office entrances, ensuring quick processing and minimizing delays while maintaining high accuracy. The system uses a combination of IR sensors, RGB cameras, and depth cameras. IR sensors, commonly used for temperature screening, are repurposed in this system to capture thermal data for identification. Thermal imaging offers robust performance in varying lighting conditions and preserves privacy by focusing on heat signatures rather than visual details. RGB cameras are included to provide detailed color and texture information, while depth cameras (e.g., LiDAR) capture three-dimensional geometric data for enhanced identification accuracy.

In our experiments, models like ResNet34 [31] and ViT [33] trained on RGB and depth data showed high accuracy but focused on irrelevant features such as clothing details or background elements in some cases. Conversely, thermal data consistently enabled the models to focus on subject-specific features, as evidenced by heatmap analyses. This highlights the potential of thermal imaging as the primary modality for person identification, with RGB and depth data serving as supplementary sources of information in a multimodal system. This approach ensures adaptability, privacy, and reliability in diverse operational scenarios.

### 4.2. Setup

**Figure 1:**
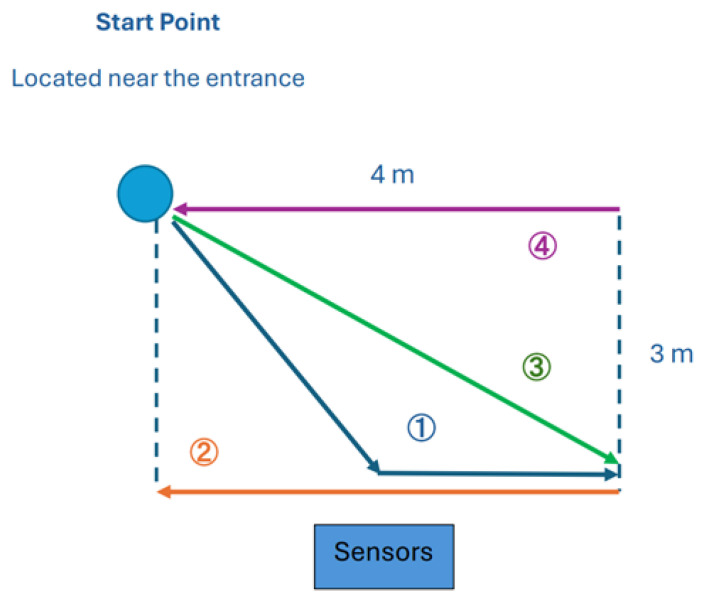
Experimental floor plan illustrating the four predefined walking paths used to evaluate the system. The arrows indicate the direction in which the subjects are walking, and the numbers indicate the walking paths. Sensors (LiDAR and FLIR) are mounted 2 m high, capturing data as participants follow the paths under different conditions. The labels are color-coded to match the corresponding walking paths depicted in this figure.

To evaluate the performance of different modalities (RGB, thermal, and depth) in person identification, we designed a controlled experimental environment simulating an office entrance. Figure 1 shows how we setup our experiments. The system consists of two sensors: a LiDAR depth camera and a FLIR thermal camera, mounted side by side on a table positioned 2 m above the ground. The horizontal distance in Figure 1 spans 4 m.

Regarding the effective distance between the subject and the sensor for the three methods discussed—RGB, thermal, and depth imaging—these distances depend on the specific sensor technologies and environmental factors. As shown in Table 2, for RGB imaging, the effective distance typically ranges from 10 to 50 m, with high-resolution cameras able to extend this range up to 100 m in well-lit conditions. However, performance can degrade in low-light environments without auxiliary lighting. Thermal imaging, using the FLIR C5 thermal camera (Teledyne FLIR, Wilsonville, OR, USA), is effective at distances of approximately 5 to 30 m, depending on environmental factors such as temperature variations and atmospheric interference. Depth imaging captured by the Intel RealSense L515 (Intel, Santa Clara, CA, USA), maintains optimal accuracy within 1 to 4 m but can extend up to 9 m for general depth data collection. The above is the theoretical effective distance. Also, we informed the effective experimental distance in Table 2. To clarify, the distances provided represent the ranges used during our experiments rather than the maximum effective distances specified by the sensor manufacturers. These distances were chosen based on the setup and constraints of our experimental environment to ensure consistent and reliable data collection across all modalities. The actual distance in our experiment does not exceed 3 m. In real-world application, we do not need such effectiveness for very far away distances.

These sensors are oriented forward to capture data from individuals walking through the test area. The test area is a rectangular region where the subjects follow predefined paths.

The experiments involved four walking scenarios (labeled 1, 2, 3, and 4) to represent different movement patterns: Scenario 1: The subject starts from the top-left corner, walks diagonally toward the sensors, stops approximately 0.5 m in front of the setup, and continues horizontally to the bottom-right corner. Scenario 2: The subject starts at the bottom-left corner and walks horizontally across the setup to the bottom-right corner. Scenario 3: The subject starts at the top-left corner and moves diagonally toward the bottom-right corner. Scenario 4: The subject starts at the top-right corner and walks horizontally across the setup to the top-left corner. Each scenario was repeated 10 times per subject. For the first five repetitions, the subjects were instructed to look directly at the sensors while walking. In the remaining five repetitions, the subjects walked naturally without focusing on the sensors.

These scenarios simulate realistic movements in an office entrance, including both frontal and non-frontal views. The setup evaluates the sensors’ ability to capture meaningful features under varying walking patterns and angles.

## 5. Proposed Method

### 5.1. Proposed Method Details

**Figure 2:**
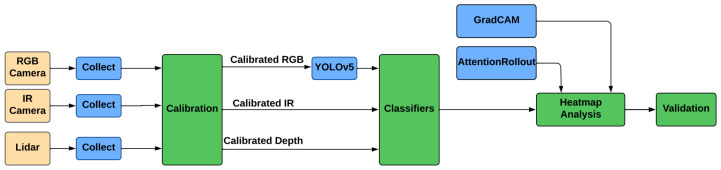
The flowchart of the proposed method in general.

The flowchart presented in Figure 2 provides a detailed visualization of the proposed method for person identification, outlining its core components: data collection, calibration, preprocessing, classification, and interpretability analysis. The scientific novelty of this method lies in its comparative study of RGB, thermal, and depth imaging, evaluating their strengths, limitations, and practical usage for person identification. This work evaluates each modality across varying resolutions, incorporates advanced interpretability techniques (GradCAM for ResNet34 and Attention Rollout for ViT), and identifies modality-specific feature reliance. The integration of these steps provides novel insights into the performance, limitations, and practical applications of these modalities, offering a foundation for developing more robust multimodal identification systems.

The process begins with data collection, where multimodal data is acquired using three distinct sensors: an RGB camera, an IR camera, and a LiDAR device. The RGB camera captures detailed visual information such as texture and color, essential for traditional person identification tasks. The IR camera collects thermal data by detecting heat signatures emitted by subjects, enabling identification in low-light or nighttime conditions. The LiDAR device generates depth data, providing three-dimensional structural information that captures the spatial features of the subject. These three modalities collectively offer complementary information for robust person identification.

Following data collection, the calibration stage ensures spatial and temporal alignment among the RGB, thermal, and depth data streams. This step is critical for achieving consistency across modalities, especially when integrating data from different sensors. Calibration facilitates precise feature extraction and enables effective fusion and analysis in later stages.

Preprocessing is performed using the YOLOv5 [23] object detection algorithm, which aims to isolate regions of interest by detecting and cropping subjects from the background. This step is intended to enhance the quality of the input data by minimizing noise and irrelevant information, thereby focusing subsequent analysis on subject-specific features. However, while YOLO performs effectively for RGB data, successfully identifying and cropping subjects with high accuracy, its performance is significantly limited for thermal and depth data. For thermal data, YOLO struggles to distinguish subjects from background heat patterns, leading to inconsistent and often inaccurate bounding boxes. Similarly, for depth data, the absence of distinct color or texture features makes it challenging for YOLO to localize subjects reliably. These limitations highlight the need for modality-specific preprocessing techniques to improve the robustness of subject detection across thermal and depth modalities.

The processed data is then fed into the classification stage, where two state-of-the-art deep learning models—Vision Transformer (ViT) and ResNet34—are utilized. ViT uses self-attention mechanisms to analyze long-range dependencies in image data, making it particularly effective for diverse modalities, including thermal imaging. ResNet34, a convolutional neural network with residual connections, extracts hierarchical features that are well-suited for RGB and depth data. Both models are trained and evaluated independently on the three modalities, enabling a comparative analysis of their performance across varying resolutions.

The final stage of the workflow involves interpretability analysis to validate the models’ decision-making processes. GradCAM [30] is employed for ResNet34 to generate heatmaps that localize the most influential regions in the input images, providing insights into the model’s focus during classification. For ViT, Attention Rollout [32] is used to trace the propagation of attention through its layers, offering a comprehensive view of the model’s focus. These interpretability techniques help uncover modality-specific patterns, such as the robust subject-focused attention in thermal data and the reliance on non-robust features in RGB and depth data.

This workflow emphasizes a systematic approach to multimodal person identification, integrating advanced sensor technologies and deep learning interpretability methods to address challenges posed by varying modalities and resolutions. By using these components, the proposed method offers a comprehensive evaluation framework for understanding the strengths and limitations of RGB, thermal, and depth imaging in diverse person identification scenarios.

#### 5.1.1. RGB-Based Identification

**Figure 3:**
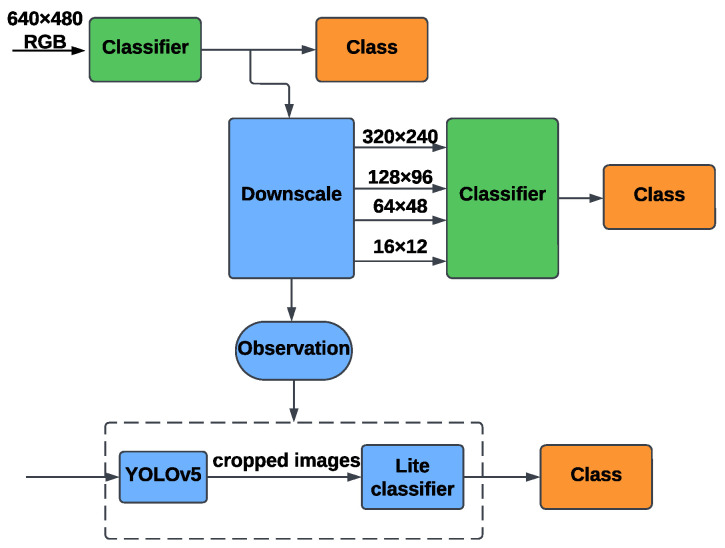
The flowchart of the RGB-based identification method.

The flowchart in Figure 3 illustrates the proposed method for RGB-based person identification, focusing on enhancements made to improve model performance and mitigate common challenges such as reliance on irrelevant features. This workflow encompasses classic image classification techniques, object detection, and various optimizations applied to state-of-the-art models.

The process begins with high-resolution RGB images captured at 640 × 480 resolution. Initially, these images are processed through a classifier to observe the baseline performance of ResNet34 and ViT models. Observations from this step revealed a tendency for the models to focus on irrelevant features, such as background textures or high-contrast details, limiting their ability to generalize effectively.

To address these issues, we introduced some modifications. In the downscaling step, RGB images are resized to progressively lower resolutions—320 × 240, 128 × 96, 64 × 48, and 16 × 12. This step enables a deeper evaluation of the models’ performance across varying spatial details, highlighting how resolution affects classification accuracy and robustness. YOLOv5 is employed to crop the region of interest, isolating the subject from the background. This preprocessing reduces noise and ensures the models focus on subject-specific features, particularly in cluttered or complex environments. The cropped images are passed through lightweight classifiers optimized for efficiency and accuracy. For ViT, this includes reducing model capacity and applying L2 regularization to minimize overfitting and improve generalization. These optimizations ensure that the models remain robust across resolutions while preventing reliance on non-informative features.

#### 5.1.2. Depth-Based Identification

**Figure 4:**
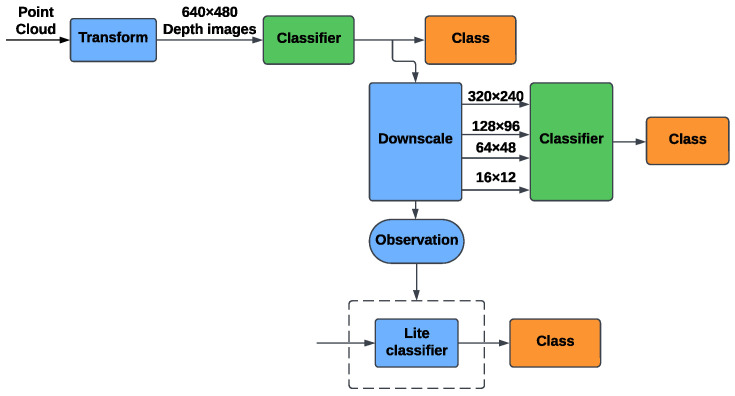
The flowchart of the Depth-based identification method.

The flowchart in Figure 4 illustrates the proposed method for depth-based person identification. This method focuses on transforming point cloud data into depth images to utilize established techniques in image processing and classification, which are well-studied and mature fields in computer vision. The workflow ensures a structured approach to handling depth data, addressing its inherent challenges while optimizing classification performance.

The process begins with point cloud data acquisition, capturing three-dimensional spatial information through LiDAR sensors. This point cloud data is converted into depth images to simplify the analysis and allow the use of image-based classification methods.

The transformed depth images are then classified using two models, ViT and ResNet34. To improve performance, specific adjustments are made to the ViT model. L2 regularization is applied during training to reduce the risk of overfitting by penalizing large weights. Additionally, the capacity of the ViT model is reduced by lowering its parameter count, which decreases computational complexity while maintaining effective learning capabilities. These changes ensure that the model remains efficient and can generalize well across depth data.

Like the RGB workflow, we also introduced a downscaling method in the depth-based identification process to evaluate the impact of resolution on model performance. Depth images, initially captured at a high resolution of 640 × 480, were progressively resized to 320 × 240, 128 × 96, 64 × 48, and 16 × 12. This step allows for a comprehensive analysis of the models’ ability to adapt to varying levels of spatial detail, especially in scenarios where high-resolution imaging may not be feasible.

#### 5.1.3. Thermal-Based Identification

**Figure 5:**
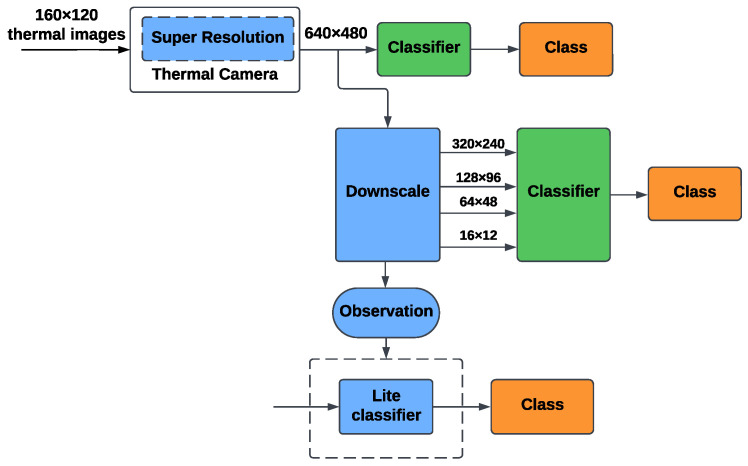
The flowchart of the thermal-based identification method.

The flowchart in Figure 5 outlines the proposed method for thermal-based person identification, incorporating the unique characteristics of the data collected by the thermal camera. The process begins with thermal images captured by the camera at an original resolution of 160 × 120. The thermal camera internally applies a super-resolution technique to increase the image resolution to 640 × 480. To evaluate the impact of resolution, the super-resolved images are downscaled to lower resolutions of 320 × 240, 128 × 96, 64 × 48, and 16 × 12. This step allows us to investigate how spatial resolution affects the classification performance of thermal data. The downscaled and original images are then fed into classification models. As with RGB-based and depth-based workflows, we also adopted the method of reducing the size of the ViT model and applying L2 regularization in the thermal-based identification process.

### 5.2. Network Details

The choice of ResNet34 and Vision Transformer (ViT) as classification models for person identification was made because of their distinct strengths and compatibility with the three data modalities—RGB, thermal, and depth. ResNet34, a well-established convolutional neural network (CNN), is chosen for its ability to extract hierarchical features efficiently. Its skip connections mitigate the vanishing gradient problem, making it well-suited for high-dimensional data such as RGB images. Additionally, ResNet34 has demonstrated robust performance in a wide range of image classification tasks, ensuring a reliable baseline for our experiments.

ViT, on the other hand, represents a cutting-edge transformer-based model that uses self-attention mechanisms to capture global dependencies in image data. This capability makes it particularly effective for modalities like thermal and depth data, where long-range spatial relationships play a crucial role in identifying individuals. However, ViT’s high capacity and computational requirements pose challenges, especially with limited datasets or lower-resolution inputs.

To address these challenges, the size of the ViT model was reduced by decreasing the number of trainable parameters and layers. This adjustment was made to prevent overfitting, as larger models tend to memorize training data rather than generalize well to unseen samples. By simplifying the architecture, aim to reduce model complexity and generalization, while ensuring better adaptability across all resolutions and modalities.

L2 regularization was added to further reduce overfitting by penalizing large weights during training. This regularization method encourages the model to learn simpler, more generalized patterns rather than overfitting to noise or irrelevant features in the data. The combination of reduced capacity and L2 regularization ensures that ViT remains lightweight, efficient, and robust across diverse datasets.

In the preprocessing stage, YOLOv5 was applied for subject detection to isolate regions of interest. While YOLO performed well for RGB images, its effectiveness was significantly limited for thermal and depth data. For RGB images, YOLO accurately identified and cropped the subject, minimizing the impact of background noise. However, in thermal data, the lack of clear contrasts between the subject and background, coupled with overlapping heat patterns, reduced YOLO’s reliability. Similarly, for depth data, YOLO struggled to produce accurate bounding boxes due to the absence of distinct features required for effective object detection. These limitations highlight the need for modality-specific preprocessing techniques, particularly for thermal and depth data.

## 6. Experimental Results

### 6.1. Experimental Settings

#### 6.1.1. Parameters

The experimental setup utilized two advanced sensing devices: the FLIR C5 thermal camera and the Intel RealSense LiDAR L515. Key specifications of these devices are summarized in Table 3. The FLIR C5 thermal camera features a 160 × 120 pixel thermal sensor and a 5-megapixel visual camera. When recording videos, the device captures thermal data at 160 × 120 resolution. However, the still images it captured can have a higher resolution, such as 640 × 480 pixels, because the FLIR C5 combines thermal data with visual details from the higher-resolution visual camera. This process, known as Multi-Spectral Dynamic Imaging (MSX), overlays visual details onto thermal images, enhancing clarity and providing more detailed images. Making it well-suited for low-light and privacy-sensitive environments. In contrast, the Intel RealSense L515 provides high-resolution RGB (1920 × 1080) and depth (1024 × 768) images, both captured at 30 FPS, utilizing LiDAR technology for precise spatial mapping. However, in this experiment, the LiDAR was configured to collect both RGB and depth data at a resolution of 640 × 480 pixels to balance spatial detail with computational efficiency and to ensure consistency across all modalities.

Both devices were mounted at a height of 2 m to simulate typical office entrance settings, ensuring an unobstructed field of view for data acquisition. The FLIR C5’s IR array sensor is used for capturing heat signatures, while the L515’s combination of RGB and depth sensors offers rich texture and geometric data. These complementary characteristics facilitate a robust comparative analysis of the RGB, thermal, and depth modalities for person identification.

#### 6.1.2. Data Set

The dataset for this study was collected using two sensors: a FLIR C5 thermal camera and an Intel RealSense L515 LiDAR sensor. The FLIR C5 captured thermal videos at 15 FPS, with each frame extracted and resized to 640 × 480 pixels to standardize the resolution. The Intel RealSense L515 simultaneously collected RGB and depth images directly at a resolution of 640 × 480 pixels and a frame rate of 30 FPS. The data was gathered from six healthy participants, including two females and four males. This multimodal data acquisition ensures the availability of thermal, RGB, and depth modalities for a comprehensive analysis of person identification.

To evaluate the effect of image resolution on person identification performance, the collected data for all three modalities (thermal, RGB, and depth) was resized into multiple resolutions: 320 × 240, 128 × 96, 64 × 48, and 16 × 12 pixels. The resizing was performed using the resize function in Python. These resized datasets enable an exploration of model robustness and performance across varying image resolutions, reflecting real-world scenarios where data quality may differ.

The dataset includes images categorized by subject identity and walking scenario, ensuring that the diversity of movement patterns is maintained. To prepare the thermal data, individual frames were extracted from the original videos. For RGB and depth data, images were extracted directly from the LiDAR sensor and resized accordingly. The dataset was split based on predefined walking scenarios to ensure consistent representation in both training and validation sets.

YOLO was applied to all three datasets—RGB, thermal, and depth—to detect and crop subjects before training the models. The detection success rates, as shown in Table 4, varied significantly across different modalities and resolutions. For RGB images, YOLO performed exceptionally well at higher resolutions, achieving a detection rate of 99.51% at 640 × 480 and 99.46% at 320 × 240. However, its performance dropped sharply to 49.75% at 64 × 48, becoming ineffective at 16 × 12.

In contrast, thermal images showed limited YOLO effectiveness, with detection rates of 5.94% at 640 × 480 and a peak of 20.62% at 320 × 240. Detection in thermal images became increasingly challenging at lower resolutions, dropping to 0% at 16 × 12. Depth images presented the greatest challenge, with minimal detection rates at higher resolutions (0.01% at 640 × 480) and marginal improvement at 64 × 48 (3.50%). YOLO failed entirely at the lowest resolution for all modalities.

The performance of YOLO as a preprocessing tool varies significantly across the three modalities, with particular challenges observed in thermal and depth data, especially at lower resolutions. For RGB data, YOLO performs reliably due to the inherent properties of RGB images, such as clear texture, color, and high contrast, which facilitate accurate object detection. This enables YOLO to effectively isolate subjects from the background, minimizing noise and improving the focus of the classification models. As a result, YOLO preprocessing significantly enhances the robustness of RGB data for person identification tasks. For thermal data, YOLO struggles to differentiate subjects from background heat patterns. This limitation stems from the increased presence of irrelevant heat signatures and diminished clarity in subject contours at lower spatial scales. However, despite these challenges, the classifiers used for thermal data are less reliant on YOLO preprocessing, as they tend to focus directly on the subject’s heat-emitting regions, such as the head and torso. This inherent property of thermal imaging allows the models to maintain robust classification performance, even when YOLO’s subject detection is less effective.

For depth data, YOLO faces significant difficulties due to the absence of texture and color features, which are critical for accurate object detection. YOLO often fails to produce reliable bounding boxes for depth images, introducing additional noise and reducing the effectiveness of depth data in person identification tasks. This shortfall highlights the need for more preprocessing methods specialized to the unique characteristics of depth imaging.

To address these challenges, future work will explore alternative or enhanced preprocessing strategies. For thermal data, integrating advanced segmentation algorithms or adaptive cropping methods could improve subject isolation. For depth data, developing specialized detection algorithms that use 3D spatial features or incorporate frame-by-frame consistency may provide more reliable solutions. Fine-tuning YOLO for depth-specific attributes or adopting lighter detection frameworks optimized for these modalities will also be investigated. These improvements aim to enhance the robustness and accuracy of object detection, particularly in challenging multimodal datasets.

On a related context, an interesting observation can be made from Table 4 regarding thermal and depth imaging. For both modalities, the detection rate seems to decrease as resolution increases. This can be attributed to several factors inherent to the nature of these modalities and their data processing workflows.

For thermal imaging, the primary reason lies in the characteristics of the original data. Thermal cameras, such as the FLIR C5 used in our study, capture data at a relatively low native resolution. Higher-resolution images are typically generated through upscaling algorithms implemented by the device manufacturer, which interpolate additional pixels to enhance the apparent resolution. When the resolution is reduced, the images approximate their original form, preserving the inherent structure of the thermal data with minimal artifacts. Conversely, higher-resolution thermal images often introduce more noise and artifacts during upscaling, increasing the complexity for classification algorithms and potentially misleading the detection process. This additional noise, combined with the increased computational load, adversely impacts the model’s ability to focus on subject-specific heat signatures.

For depth imaging, a similar trend is observed due to noise and complexity. Depth data is inherently constrained by the resolution of the Lidar point cloud, which defines the spatial granularity of the depth map. Lower-resolution depth images more faithfully represent the original point cloud data by reducing the reliance on interpolation to fill in missing pixels. At higher resolutions, the interpolation algorithms used to enhance depth data can introduce inaccuracies and artificial features, complicating the classification task. These interpolated features may not correspond to the actual physical structures, leading to reduced detection accuracy and increased susceptibility to noise.

**Figure 6:**
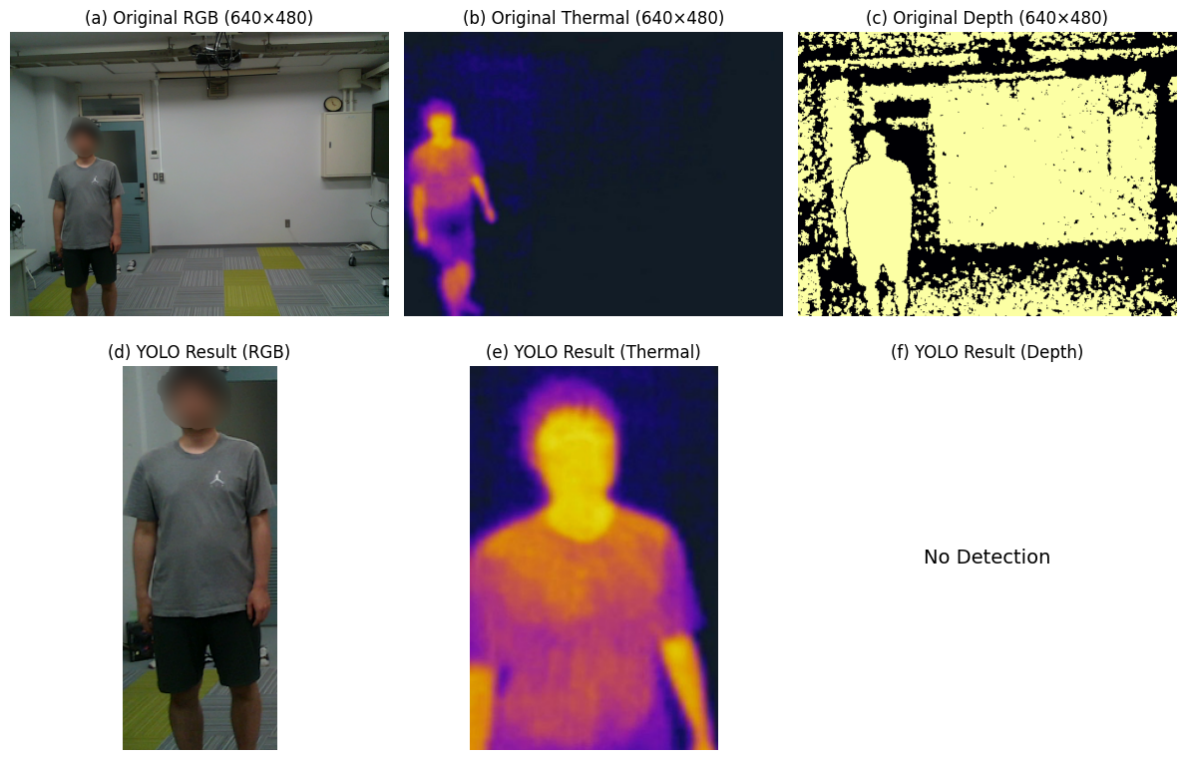
Examples of RGB, thermal, and depth images collected at 640 × 480 resolution, along with YOLO-based subject detection results. The first row illustrates the original images: (**a**) RGB image, (**b**) thermal image, and (**c**) depth image. The second row shows the YOLO detection results: (**d**) subject detected in the RGB image, (**e**) subject detected in the thermal image, and (**f**) “No Detection” for the depth image.

Figure 6 illustrates examples of RGB, thermal, and depth images collected at a resolution of 640 × 480 pixels, along with the corresponding results of YOLO-based subject detection for RGB and thermal modalities. The first row (sub-figures (a), (b), and (c)) shows the original images for each modality: sub-figure (a) displays the RGB image captured by the Intel RealSense LiDAR L515, sub-figure (b) presents the thermal image extracted from the FLIR C5 camera, and sub-figure (c) shows the depth image generated by the LiDAR sensor. These examples highlight the diverse characteristics of each modality, such as color and texture information in RGB, heat signatures in thermal, and geometric data in depth.

The second row (sub-figures (d), (e), and (f)) demonstrates the results of subject detection using YOLO. Sub-figure (d) shows the cropped subject detected in the RGB image, and sub-figure (e) presents the corresponding YOLO result for the thermal image. Sub-figure (f) represents the detection result for the depth image, where YOLO was unable to detect the subject effectively, resulting in “No Detection”. This figure highlights the performance differences of YOLO across modalities, highlighting its effectiveness for RGB and thermal data while demonstrating limitations for depth data.

#### 6.1.3. Evaluation Metrics

The evaluation of the proposed person identification method is conducted using three key metrics: accuracy, confusion matrix, and heatmap analysis. These metrics collectively provide a comprehensive assessment of the models’ performance and interpretability across RGB, thermal, and depth modalities.

Accuracy is used as the primary metric to quantify the overall performance of the models. It is defined as the proportion of correctly identified samples out of the total samples in the dataset. This metric provides a direct measure of the identification success rate for each modality and model.

Confusion matrices are utilized to analyze the distribution of predictions across different classes. These matrices provide insights into the models’ tendencies to misclassify specific classes, allowing for a deeper understanding of their performance on each modality. Patterns observed in the confusion matrices highlighte potential biases or challenges in distinguishing particular classes.

Heatmap analysis is conducted to interpret the decision-making process of the models. For ResNet34, GradCAM is used to generate heatmaps that visualize the regions of input images that most influence the model’s predictions. Similarly, Attention Rollout is applied to the ViT to trace how attention propagated through the model’s layers. These interpretability tools help determine whether the models focus on relevant subject features or are distracted by irrelevant details such as the background or clothing.

Together, these metrics provide a detailed evaluation of the models’ identification performance and interpretability, enabling a comparative analysis across RGB, thermal, and depth modalities.

### 6.2. Experimental Results and Analysis

#### 6.2.1. Experimental Results

**Figure 7:**
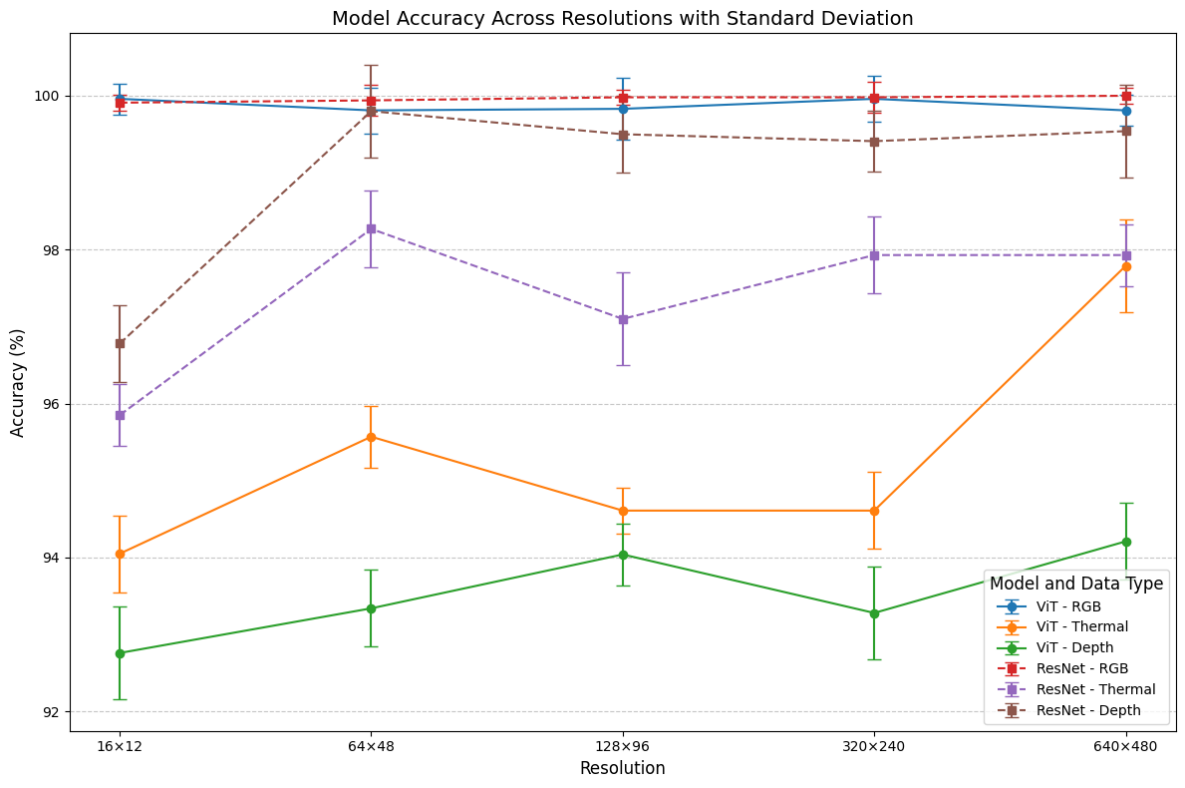
Accuracy performance of ViT and ResNet34 across different resolutions (16 × 12, 64 × 48, 128 × 96, 320 × 240, and 640 × 480) and modalities (RGB, thermal, and depth images).

In this section, we present a comprehensive analysis of the performance of ViT and ResNet34 models across three data modalities—RGB, thermal, and depth images—at varying resolutions (16 × 12, 64 × 48, 128 × 96, 320 × 240, and 640 × 480). Table 5 shows the identification accuracy of different configurations. The evaluation focuses on accuracy trends across different configurations, as illustrated in the line chart (Figure 7). The error bars in this figure represent the standard deviation of accuracy across different classes, providing additional insights into the model’s consistency. Additionally, we analyze the training and validation behaviors through learning curves and utilize heatmaps to interpret the focus areas of the models during inference. This comprehensive evaluation aims to uncover the strengths and limitations of each model and modality, offering valuable insights into their applicability for person identification tasks.

Both models consistently demonstrate high accuracy for RGB data across all resolutions, with ResNet34 achieving near-perfect accuracy at 640 × 480 resolution. The experiment results reveal that thermal and depth data exhibit a noticeable drop in accuracy as the resolution decreases, with both modalities achieving their lowest accuracy at the smallest resolution of 16 × 12. Interestingly, this trend is not linear, as the accuracy for both modalities except the ViT-RGB set shows an unexpected increase at the 64 × 48 resolution. This unusual phenomenon can likely be attributed to the intricate interplay between image resolution and the model’s attention mechanisms, which adapt differently across varying levels of detail and feature density.

When the resolution increases, the models are required to process a significantly larger number of pixels, which can dilute the focus of attention mechanisms like those in ViT and ResNet34. As a result, these models may struggle to identify patterns that are distributed across a larger spatial domain. This challenge is particularly pronounced in modalities like thermal and depth, where the patterns of interest (e.g., heat signatures or geometric structures) are inherently subtler or less distinct than those in RGB images. At resolutions like 64 × 48, the balance between pixel density and the model’s capacity to focus on meaningful regions enables better recognition of patterns, explaining the improved performance compared to other resolutions.

**Figure 8:**
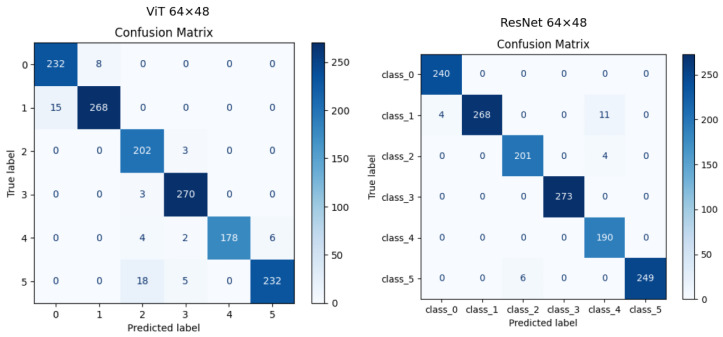
Combined confusion matrices of ViT and ResNet models trained on thermal images with a resolution of 64 × 48. The left matrix corresponds to the ViT model with an overall accuracy of 95.57%, while the right matrix represents the ResNet model with an overall accuracy of 98.27%. Both matrices provide insights into the classification performance across six classes, highlighting the strengths and limitations of each model in handling thermal data at this resolution.

Figure 8 shows the confusion matrices for the ViT and ResNet models, based on thermal images with a resolution of 64 × 48. It reveals critical insights into their classification performance and highlights the strengths and limitations of each approach. Both models demonstrate high accuracy across most classes, but notable differences emerge in their ability to handle inter-class ambiguities and extract meaningful features from low-resolution thermal data.

For the ViT model, the confusion matrix indicates strong overall performance, with high accuracy for the majority of classes. However, specific challenges arise in distinguishing between certain classes. For instance, class 1 exhibits misclassification with class 0, where 15 samples are incorrectly predicted. This suggests that the ViT model struggles to differentiate thermal patterns with overlapping characteristics, likely due to the global nature of its attention mechanism. Additionally, a notable issue is observed in class 5, where 18 samples are misclassified as class 2, further highlighting the difficulty of extracting distinct features for these categories. Despite these challenges, the ViT model performs better in identifying class 3 and class 0 with minimal misclassification, indicating that its attention mechanism can effectively use distinct features in certain cases.

The ResNet model demonstrates superior classification performance compared to the ViT model, particularly in minimizing misclassifications for challenging classes. The confusion matrix shows near-perfect classification for class 0 and class 5, underscoring ResNet’s ability to effectively handle thermal patterns even at low resolution. However, minor confusion is observed in class 1, where 11 samples are misclassified as class 4, potentially due to overlapping thermal signatures. ResNet’s convolutional architecture, which hierarchically extracts features, appears better suited for distinguishing fine-grained differences in thermal data.

When comparing the two models, the ViT model exhibits more spread errors across multiple classes, reflecting its reliance on global attention mechanisms that may struggle with subtle inter-class distinctions. In contrast, the ResNet model demonstrates more localized misclassification trends, attributed to its convolutional feature extraction approach. While the ViT model performs better in specific cases with highly distinctive features, the ResNet model shows better generalization and overall accuracy at this resolution.

Both models face challenges due to the limitations of low-resolution thermal data, as the reduced spatial information hinders the ability to extract meaningful patterns. This is particularly evident in the ViT model, which requires larger, distinct regions for effective attention. The ResNet model, while more robust, also shows signs of degradation in performance for overlapping classes.

Although this study thoroughly evaluated the efficacy of RGB, thermal, and depth modalities for person identification across six classes, we recognize that the inclusion of more subjects would enhance the generalizability and robustness of the findings. Due to resource constraints during the data collection phase, the dataset was limited to six classes. Future work will aim to expand the dataset to include more subjects and further validate the proposed approach.

#### 6.2.2. Performance Analysis of ViT Model

**Figure 9:**
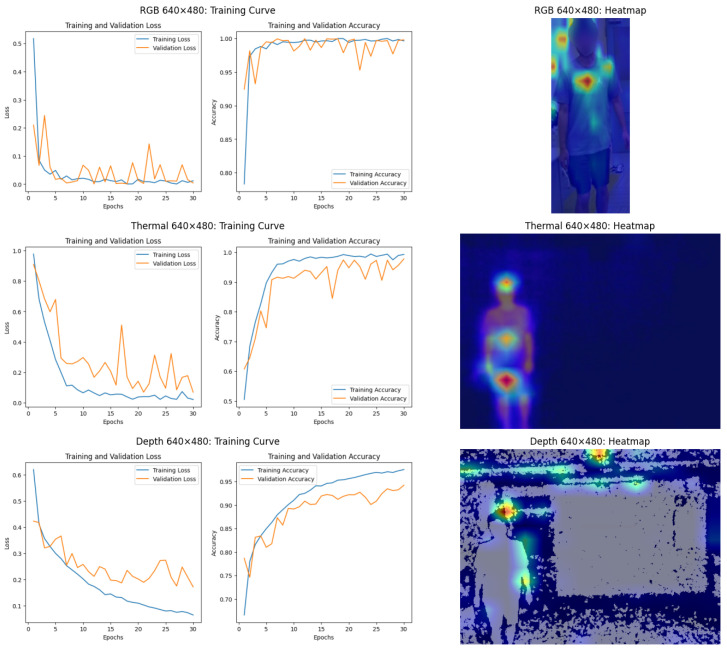
Training curves and heatmaps for RGB, thermal, and depth data at a resolution of 640 × 480 using the ViT model. The left column shows the training and validation loss and accuracy, while the right column presents heatmaps of the model’s focus.

**Figure 10:**
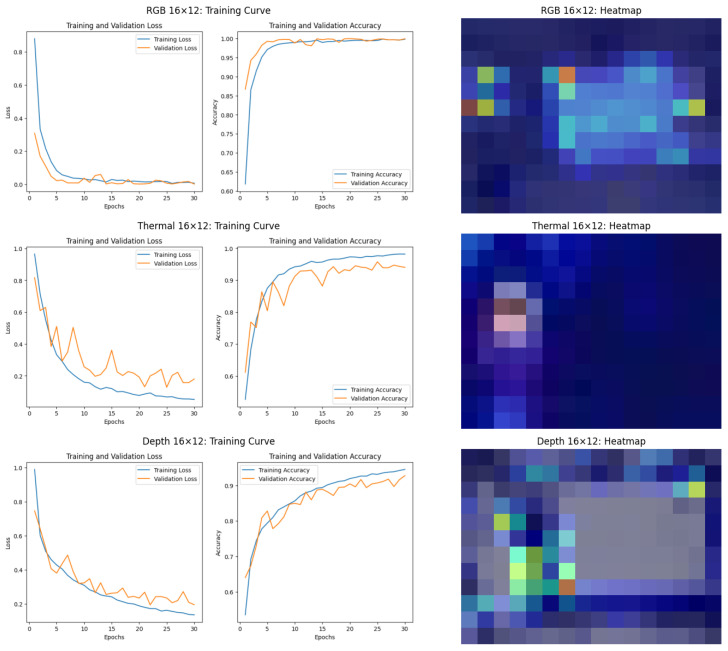
Training curves and heatmaps for RGB, thermal, and depth data at a resolution of 16 × 12 using the ViT model. The left column shows the training and validation loss and accuracy, while the right column presents heatmaps of the model’s focus.

The analysis presented in this section focuses exclusively on results obtained using ViT models for three different data modalities: RGB, thermal, and depth. Figure 9 and Figure 10 present the performance of these models at 640 × 480 and 16 × 12 resolutions, respectively. These figures illustrate both the training and validation loss and accuracy and the corresponding heatmaps, which highlight the areas of focus during classification. By examining these results, valuable insights are provided into the strengths and limitations of ViT models when applied to person identification tasks across varying resolutions and data modalities.

The training curves and corresponding heatmaps in Figure 9 present the performance of ViT models on high-resolution data (640 × 480). For RGB data, the training and validation curves show minimal divergence, with accuracy approaching 100%. However, the heatmaps reveal that the model often focuses on background elements and clothing logos, relying on non-robust features, which limits generalization. Thermal data demonstrates exceptional robustness at 640 × 480 resolution. The training and validation losses align closely, and heatmaps confirm that the model focuses on the subject’s body, particularly areas emitting heat, enabling consistent performance. For depth data, a gap between training and validation accuracies indicates overfitting. Heatmaps show dispersed attention across the subject and background, underscoring the challenge of isolating meaningful features from depth images.

At the 16 × 12 resolution, the limitations of thermal and depth data are pronounced, as seen in Figure 10. For RGB images, the training and validation losses are very similar, and their corresponding accuracies align closely. This indicates that the ViT model generalizes well on RGB data at the 16 × 12 resolution, despite the challenges of low resolution. There is no significant divergence between the training and validation curves, suggesting minimal overfitting. However, the corresponding heatmap reveals limitations in the attention mechanism. The attention is scattered across irrelevant regions, including the background and non-distinctive areas of the subject. This indicates that at such low resolution, the model struggles to focus on meaningful features, potentially due to the lack of fine-grained details in the data.

For the thermal data in 16 × 12 resolution, the training loss decreases consistently, but the validation loss exhibits greater oscillations. While the validation accuracy remains high, the gap between the training and validation losses indicates that the model might be overfitting slightly or struggling to generalize perfectly at this resolution. However, the heatmap shows concentrated attention on the subject’s thermal signature, primarily focusing on the head and torso regions. This indicates that the overfitting might not come from the model attending to irrelevant features (e.g., background), as the heatmap suggests the model is concentrating on the subject’s key thermal regions. Instead, the overfitting could result from the limited variability in the dataset or the inherent challenges of low-resolution thermal data. This lack of diversity might cause the model to memorize specific patterns in the training set that do not generalize well to the validation set. In practical terms, this means the model is learning the correct regions of interest. However, it might not be robust to variations in the thermal data (e.g., different postures).

For the depth data in 16 × 12 resolution, the divergence is pronounced too, with the validation loss and accuracy plateauing at lower levels compared to the training metrics. This suggests that the ViT model struggles to generalize effectively with depth data at this resolution. The heatmap further supports this observation, showing scattered and inconsistent attention patterns. The model could not focus on meaningful features, highlighting the challenge of using depth data for person identification tasks at extremely low resolutions.

#### 6.2.3. Performance Analysis of ResNet34 Model

**Figure 11:**
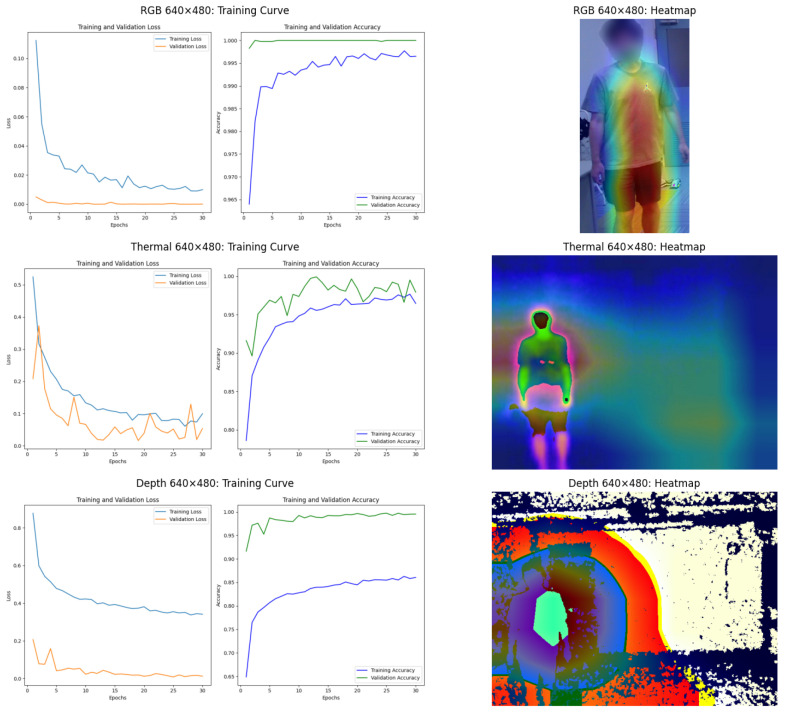
Training curves and heatmaps for RGB, thermal, and depth data at a resolution of 640 × 480 using the ResNet34 model. The left column shows the training and validation loss and accuracy, while the right column presents heatmaps of the model’s focus.

**Figure 12:**
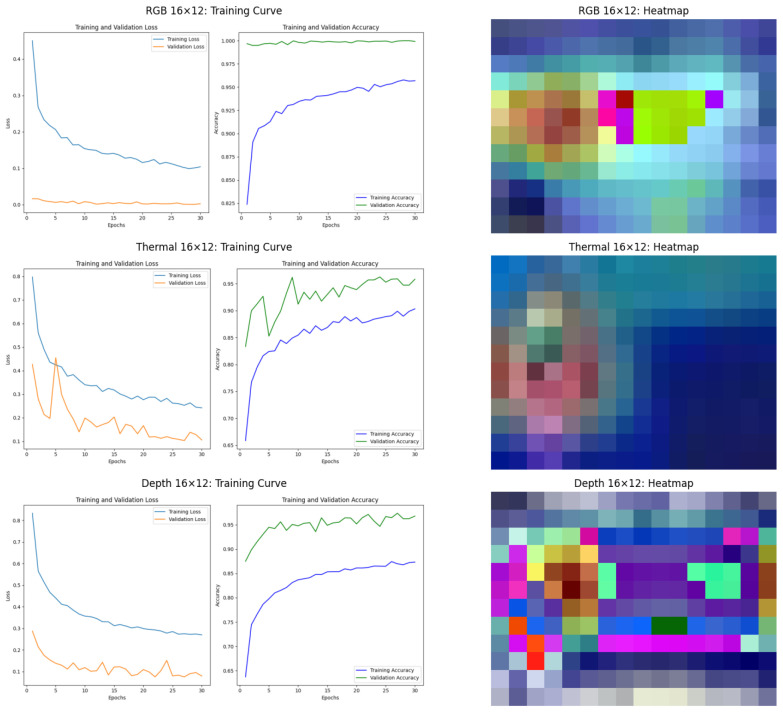
Training curves and heatmaps for RGB, thermal, and depth data at a resolution of 16 × 12 using the ResNet34 model. The left column shows the training and validation loss and accuracy, while the right column presents heatmaps of the model’s focus.

The ResNet34 model results, presented in Figure 11 and Figure 12, align with the trends observed for the ViT model but exhibit some differences. For RGB data at 640 × 480 resolution, the ResNet34 model exhibits minimal overfitting, as indicated by the convergence of training and validation losses. The model achieves high accuracy, demonstrating effective learning from the data. However, GradCAM heatmaps reveal a limitation: the model’s attention is concentrated on the subject’s clothing area, which might include logos or patterns, rather than focusing exclusively on identity-relevant features. This reliance on non-robust features could limit the model’s generalization capability in scenarios with varying clothing or occlusions. Thermal data maintains strong generalization across both resolutions for ResNet34. At 640 × 480 resolution, heatmaps highlight attention on critical areas like the head and torso, ensuring reliable classification. For depth data at 640 × 480 resolution, while the training and validation losses appear to converge, the model begins to show signs of underfitting. This is evident from the relatively slower increase in training accuracy and the lack of further improvement in validation accuracy. Moreover, the heatmap analysis reveals that the model’s attention is not exclusively focused on the subject’s body. Instead, the attention spreads to areas outside the subject, including the background.

At 16 × 12 resolution, the ResNet34 model maintains high validation accuracy but appears underfitting in RGB data. The training curve shows the validation set does not show the same convergence trend as the training set. For RGB and depth data, the GradCAM heatmaps highlight a significant concern: the model’s attention extends to irrelevant features, such as background areas, instead of focusing solely on the subject. This behavior suggests that at lower resolutions, the model struggles to differentiate between relevant and irrelevant features. For the thermal data in this resolution, the model remains robust, with heatmaps focused on the subject’s core regions, demonstrating the strength of thermal imaging.

### 6.3. Discussion

This section makes a comprehensive comparison of the ViT and ResNet34 models for person identification across three data modalities: RGB, thermal, and depth. It examines the strengths and limitations of each modality, highlighting the impact of resolution and architectural differences on performance. The analysis incorporates insights from training curves and heatmaps generated using GradCAM for ResNet34 and Attention Rollout for ViT, focusing on the models’ attention mechanisms and their reliance on salient features versus background elements. Additionally, the role of preprocessing, particularly YOLO-based cropping, is critically evaluated, acknowledging its benefits and limitations in isolating subjects from the background.

#### 6.3.1. RGB Data: Challenges with Background Dependency

When applied to RGB data, both ViT and ResNet34 encounter significant challenges, particularly with low-resolution inputs such as 16 × 12. The Attention Rollout heatmaps for ViT demonstrate that the model frequently focuses on non-relevant background textures or high-contrast details, such as logos on clothing, rather than consistently identifying the subject. This reliance on background or high-contrast features diminishes the model’s generalization ability, especially as spatial details become less pronounced at lower resolutions. The sharp decline in accuracy with lower-resolution RGB data reflects the model’s dependence on fine-grained texture and color information, which are largely absent in such scenarios.

ResNet34 also struggles with RGB data but exhibits slightly better generalization compared to ViT. The training curves indicate a smaller gap between training and validation accuracy, suggesting less overfitting. GradCAM heatmaps for ResNet34, however, show attention on irrelevant features in the background, particularly in cluttered environments, which can confuse the model. To address these limitations, YOLO-based cropping was applied as a preprocessing step to isolate the subject and reduce the influence of background noise. While this method helps in some cases, it does not consistently improve results in complex or cluttered backgrounds where YOLO fails to accurately predict bounding boxes. This limitation underscores the need for more robust subject detection mechanisms or advanced preprocessing techniques to enhance the performance of RGB-based models.

#### 6.3.2. Thermal Data: A Robust Modality

Thermal imaging demonstrates significant robustness for both ViT and ResNet34 models. For ViT, the alignment between training and validation metrics suggests strong generalization in high resolution but starts overfitting at low resolution. The Attention Rollout heatmaps consistently show that the model focuses on the subject’s thermal signature, avoiding distractions from the environment. This performance highlights the advantages of thermal data, where distinct subject features stand out regardless of environmental conditions.

ResNet34 also performs well with thermal data, maintaining close alignment between training and validation curves. However, at low resolutions (e.g., 16 × 12), the model shows a slight tendency toward underfitting. GradCAM heatmaps for ResNet34 confirm its ability to use the subject’s thermal features effectively, although occasional attention to edges or noise in the background is observed. These results affirm thermal imaging’s potential for person identification, particularly in low-light or visually cluttered environments. The reliance on YOLO cropping is less critical for thermal data due to its inherent clarity in isolating subjects from the background.

#### 6.3.3. Depth Data: Sensitivity to Resolution

Depth data presents unique challenges for both models, particularly at lower resolutions. For ViT, overfitting becomes more pronounced, as evidenced by a significant gap between training and validation accuracy. Attention Rollout heatmaps reveal inconsistent attention patterns, with the model focusing on depth artifacts or uniform regions instead of the subject. This behavior highlights the difficulty of extracting meaningful features from depth data, especially when resolution constraints exacerbate noise and artifacts.

ResNet34, in contrast, demonstrates better generalization with depth data at higher resolutions. However, at 16 × 12 resolution, the model struggles with noise and artifacts, leading to reduced performance. GradCAM heatmaps show scattered attention, with the model occasionally misinterpreting background noise as relevant features. YOLO cropping partially mitigates these issues, but its performance is often unreliable with depth data due to the absence of distinct texture or color features required for precise subject detection. These challenges suggest that future work should focus on improving preprocessing techniques for depth data, such as denoising and artifact removal, and developing specialized detection methods for depth images.

#### 6.3.4. Heatmap Analysis: GradCAM and Attention Rollout

The heatmaps generated by GradCAM for ResNet34 and Attention Rollout for ViT reveal critical insights into how the models process RGB, thermal, and depth data, shedding light on their strengths and limitations across different scenarios. While these visualizations enhance interpretability, they also highlight significant challenges, particularly with RGB and depth data.

For RGB data, both GradCAM and Attention Rollout heatmaps demonstrate a tendency for the models to focus on irrelevant background details, such as textures, high-contrast elements, or logos on clothing. This reliance on non-informative features hampers the models’ ability to consistently identify the subject, especially in cluttered environments. YOLO cropping is applied as a preprocessing step to isolate the subject and mitigate background interference, but its effectiveness is limited in cases where bounding box predictions are inaccurate or overlap with background regions. The Attention Rollout heatmaps for ViT show a broad, scattered pattern of attention, with focus spread across multiple regions, suggesting an imprecise emphasis. GradCAM heatmaps for ResNet34, while more localized, still include background elements, reflecting persistent challenges in effectively using RGB data.

For thermal data, both interpretability methods consistently highlight focused attention on the subject’s thermal signature, specifically on key areas such as the head, chest, and limbs. This concentration on meaningful features underscores the robustness of thermal imaging, which is less influenced by environmental clutter or reduced spatial details. Even across varying resolutions, the heatmaps demonstrate that thermal data reliably isolate person-specific features, enabling both ViT and ResNet34 to maintain strong generalization. This robustness makes thermal data particularly advantageous in scenarios with complex backgrounds or low-resolution constraints.

The depth data heatmaps, however, reveal substantial limitations. Both GradCAM and Attention Rollout highlight scattered and inconsistent attention patterns, with the models often focusing on artifacts, uniform regions, or noisy areas instead of the subject. These findings reflect the inherent challenges of depth data, which lack the clear, high-contrast features found in RGB and thermal modalities. YOLO preprocessing proves less effective for depth data due to the absence of distinct regions necessary for accurate bounding box predictions. As a result, the models struggle to consistently isolate meaningful features, particularly at lower resolutions where depth noise and artifacts are more pronounced.

These heatmap analyses underline the importance of modality-specific challenges and suggest that future work should focus on enhancing interpretability and preprocessing techniques. For RGB data, advanced subject detection algorithms or attention regularization during training could help models focus on relevant regions. For depth data, denoising and feature enhancement methods might improve reliability, while thermal imaging remains a robust modality for person identification under diverse conditions.

#### 6.3.5. Multi-Modal Analysis

One limitation of this study is the lack of specialized model architectures explicitly adapted for multi-modal data. The current work evaluates the performance of RGB, thermal, and depth data independently to identify their strengths and limitations, but does not integrate these modalities into a unified framework. While this approach provides valuable insights into modality-specific performance, it misses the opportunity to exploit the complementary nature of these modalities.

Multi-modal integration could significantly enhance person identification performance by combining the strengths of each modality. For example, thermal data provide robust subject-specific features under low-light conditions, RGB data capture rich texture and color information in well-lit environments, and depth data offer three-dimensional structural details. A specialized multi-modal architecture could utilize shared and modality-specific branches to extract features unique to each modality while learning a shared representation for classification. For example, the late fusion combines modality-specific features at a later stage. Dynamic attention mechanisms adaptively weigh each modality’s contribution based on context. Both of them could further improve the robustness of the system.

Future work will focus on developing such multi-modal frameworks and incorporating preprocessing techniques to each modality. By addressing the limitations of the current study, these efforts aim to unlock the full potential of multi-modal data for person identification tasks, bridging the gap between modality-specific insights and practical, integrated solutions.

## 7. Conclusions

This study explored the performance of ViT and ResNet34 models for person identification using three distinct data modalities: RGB, thermal, and depth. Across varying resolutions, the results highlighted the strengths and limitations of each modality, providing valuable insights into the behavior of these models under different conditions. Thermal data consistently demonstrated superior robustness and reliability, with both models effectively taking advantage of the distinct thermal signatures of subjects to achieve strong generalization. RGB data, while performing well at higher resolutions, suffered from background dependency, especially at lower resolutions. Depth data, although promising at higher resolutions, exhibited significant performance degradation at lower spatial scales due to scattered and inconsistent attention patterns.

The heatmaps generated through GradCAM (for ResNet34) and Attention Rollout (for ViT) provided deeper insights into the attention mechanisms of the models. These visualizations revealed that while thermal data allowed both models to focus on meaningful features, RGB and depth data were often influenced by irrelevant background details or artifacts. Preprocessing steps, such as YOLO-based cropping, were introduced to mitigate these issues; however, their effectiveness was limited, particularly for cluttered environments or depth data.

Looking forward, several directions can be pursued to enhance the robustness and accuracy of person identification systems. First, integrating multiple modalities, such as RGB, thermal, and depth, could make use of the complementary strengths of each data type. Thermal data, for instance, could compensate for the limitations of RGB and depth modalities, especially in low-light or complex backgrounds. Second, advanced preprocessing techniques, including segmentation-based methods, could further refine the regions of interest and reduce the impact of irrelevant features. Third, exploring hybrid architectures that incorporate modality-specific feature extractors or attention mechanisms could optimize performance for each data type.

Additionally, incorporating regularization techniques and data augmentation strategies could help address overfitting. Future work could also focus on enhancing the interpretability of models by developing more advanced visualization techniques that extend beyond GradCAM and Attention Rollout. Finally, expanding the scope of experiments to include more diverse datasets and real-world scenarios will be critical in validating the generalizability of the proposed methods.

In conclusion, this study highlighted the potential of thermal imaging as a robust modality for person identification while also highlighting the challenges and opportunities associated with RGB and depth data. By addressing these challenges and using the insights gained, future research can advance the development of reliable and adaptable person identification systems for a wide range of applications. 

## Figures and Tables

**Table 1:** Comparison of Advantages and Disadvantages of Millimeter Wave Radar, RGB, Thermal, and Depth Data for Person Identification.

Modality	Advantages	Disadvantages
Millimeter Wave Radar	- Works in occluded environments (e.g., fog, smoke, through walls). - Resilient to lighting conditions and appearance changes.	- Limited spatial resolution. - Susceptible to electromagnetic interference. - Less effective at distinguishing fine details.
RGB	- High accuracy in well-lit conditions. - Rich texture and color information.	- Poor performance in low-light or occluded conditions. - Sensitive to changes in appearance and cluttered backgrounds.
Thermal	- Effective in low-light or nighttime scenarios. - Captures heat signatures, ensuring privacy.	- Overlapping thermal profiles reduce accuracy. - Background heat artifacts can interfere.
Depth	- Captures 3D structural features. - Relatively resilient to surface appearance changes.	- Prone to artifacts and incomplete data. - Less effective in dynamic or cluttered environments. - Noise increases at lower resolutions.

**Table 2:** Effective Distances for Data Collection Using RGB, Thermal, and Depth Imaging.

Modality	Theoretical Effective Distance Range	Experimental Effective Distance Range	Factors Influencing Effectiveness
RGB Imaging	10 to 50 m (up to 100 m with high-resolution cameras in well-lit conditions)	Up to 3 m	Performance degrades in low-light conditions without auxiliary lighting.
Thermal Imaging (FLIR C5)	5 to 30 m	Up to 3 m	Environmental factors such as temperature variations and atmospheric interference.
Depth Imaging (Intel RealSense L515)	1 to 4 m (optimal); up to 9 m (general depth data collection)	Up to 3 m	Reduced accuracy beyond 4 m; noise increases with distance.

**Table 3:** Specifications of the FLIR C5 and Intel RealSense LiDAR L515 cameras used in the experiments.

Parameter	FLIR C5 (Thermal Camera)	Intel RealSense LiDAR L515
Resolution	160 × 120 pixels	RGB: 640 × 480 pixels; Depth: 640 × 480 pixels
Frame Rate	15 FPS	RGB: 30 FPS; Depth: 30 FPS
Sensor Type	Infrared Array Sensor	LiDAR with RGB and Depth Sensors
Data Output	Thermal Videos	RGB and Depth Images
Mounting Height	2 m	2 m
Field of View (FOV)	54° × 42°	RGB: 70° × 43°; Depth: 70° × 55°

**Table 4:** YOLO Detection Rates Across Resolutions for RGB, Thermal, and Depth Data.

Resolution	RGB (%)	Thermal (%)	Depth (%)
16 × 12	0.00	0.00	0.00
64 × 48	49.75	5.07	3.50
128 × 96	98.76	15.18	0.41
320 × 240	99.46	20.62	0.07
640 × 480	99.51	5.94	0.01

**Table 5:** Identification Accuracy (%) Across Resolutions for ViT and ResNet Models.

Resolution	ViT RGB	ViT Thermal	ViT Depth	ResNet RGB	ResNet Thermal	ResNet Depth
16 × 12	99.96	94.05	92.76	99.91	95.85	96.78
64 × 48	99.81	95.57	93.34	99.94	98.27	99.80
128 × 96	99.83	94.61	94.04	99.98	97.10	99.50
320 × 240	99.96	94.61	93.28	99.98	97.93	99.41
640 × 480	99.81	97.79	94.21	100.0	97.93	99.54

## Data Availability

The data presented in this study are available on request from the corresponding author due to privacy issues.
